# Highly Sensitive E-Textile Strain Sensors Enhanced by Geometrical Treatment for Human Monitoring

**DOI:** 10.3390/s20082383

**Published:** 2020-04-22

**Authors:** Chi Cuong Vu, Jooyong Kim

**Affiliations:** Department of Organic Materials and Fiber Engineering, Soongsil University, Seoul 156-743, Korea; cuongvc287@gmail.com

**Keywords:** e-textiles, single-walled carbon nanotubes (SWCNT), silver paste, structure, shape

## Abstract

Electronic textiles, also known as smart textiles or smart fabrics, are one of the best form factors that enable electronics to be embedded in them, presenting physical flexibility and sizes that cannot be achieved with other existing electronic manufacturing techniques. As part of smart textiles, e-sensors for human movement monitoring have attracted tremendous interest from researchers in recent years. Although there have been outstanding developments, smart e-textile sensors still present significant challenges in sensitivity, accuracy, durability, and manufacturing efficiency. This study proposes a two-step approach (from structure layers and shape) to actively enhance the performance of e-textile strain sensors and improve manufacturing ability for the industry. Indeed, the fabricated strain sensors based on the silver paste/single-walled carbon nanotube (SWCNT) layers and buffer cutting lines have fast response time, low hysteresis, and are six times more sensitive than SWCNT sensors alone. The e-textile sensors are integrated on a glove for monitoring the angle of finger motions. Interestingly, by attaching the sensor to the skin of the neck, the pharynx motions when speaking, coughing, and swallowing exhibited obvious and consistent signals. This research highlights the effect of the shapes and structures of e-textile strain sensors in the operation of a wearable e-textile system. This work also is intended as a starting point that will shape the standardization of strain fabric sensors in different applications.

## 1. Introduction

Strain textile sensors enable close fitting on curvilinear surfaces or direct attachment to clothes that are very important in the new generation of portable and wearable electronics [1,2,3]. One of the critical applications is in the motion-sensing device that can detect human movements or human–machine interfaces for robotics, through the conversion of various stimuli from the human body to electrical signals. Based on that principle, most of the operating mechanisms of strain textile sensors are based on a relationship between physical quantity, stretchability, and electrical properties such as resistance or the capacitance of constituent conductive materials [4,5,6,7]. There are many fabrication methods of strain textile sensors such as coating of conductive materials on fibers, yarns, or fabrics; using conducting fibrous structures; spinning composites or coaxial fibers; and geometrical manipulation of yarns (e.g., buckling, coiling, and knitting) [8,9,10,11,12]. 

For example, Tolvanen et al. [13] discussed a stretchable and washable strain sensor based on a cracking structure for human motion monitoring. Zheng et al. [14] presented a highly stretchable and stable strain sensor from hybrid conductive composites (carbon nanofillers/polydimethylsiloxane) for large movements. Cai et al. [15] fabricated flexible strain sensors by coating and reducing graphene oxide (GO) on the surface of nylon/polyurethane (PU) fabric. Sadeqi et al. [16] developed a facile and low-cost method for the fabrication of a strain sensor based on polybutylene terephthalate (PBT) puffy thread, carbon resistive ink, and polymer coating polydimethylsiloxane (PDMS). Chen et al. [17] prepared a PPy-coated stretchable textile via a low-temperature interfacial polymerization method to function as a sensitive sensor. Many other studies on stretch sensors [18,19,20,21,22] were conclusively proven to have highly sensitive materials suitable for wearable devices. However, all of the above studies pay particular attention to complex materials or fabrication technologies that will increase the difficulty in the manufacturing process, cost, and ability in mass production. 

**Figure 1:**
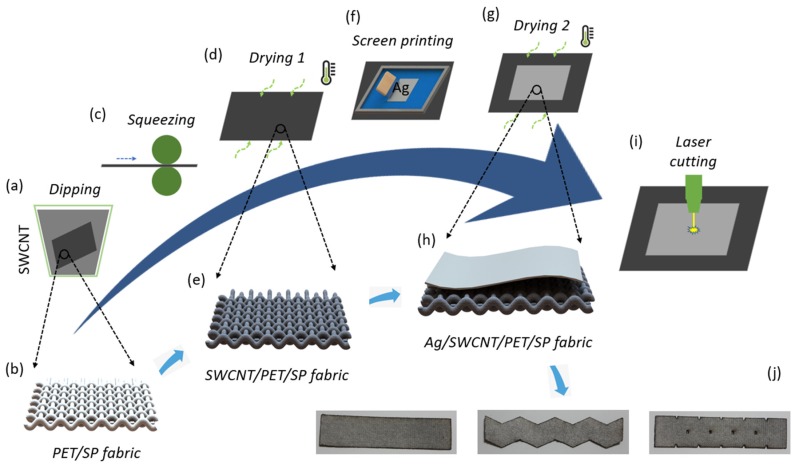
Manufacturing process from polyester/spandex (PET/SP) fabric consisting of (**a**) dipping single-walled carbon nanotubes (SWCNTs), (**b**) PET/SP fabrics, (**c**) squeezing, (**d**) drying SWCNT, (**e**) SWCNT fabrics, (**f**) screen printing Ag pastes, (**g**) drying Ag/SWCNT, (**h**) Ag/SWCNT fabrics, (**i**) laser cutting, (**j**) the different shapes of Ag/SWCNT sensors.

This research proposes a two-step approach, not only in fabrication but also in the optimal performance of the e-textile strain sensors. In the first step, we considered the structure of sensor materials. A combination of Ag pastes/SWCNTs addresses the issues of poor gauge factor (GF) at the low percentage of tension [14,15,16,17] and high hysteresis [13,22]. In the second step, through many experiments, we focused on the shape of the sensors. Changes in the shapes are the best solution to sharply increase the sensitivity of sensors without increasing the costs (as described in Section 3.2). Notably, the scalable batch manufacturing process, as shown in Figure 1, is also a highlight in view of the industrial applications because CNT-impregnated textiles and screen printing technologies are easy to approach and apply. Otherwise, the laser cutting process allows for the rapid, reliable, and scalable production of large sensor numbers.

## 2. Materials and Methods

The fabric used is polyester/spandex (PET/SP) with a ratio of 76% polyethylene terephthalate and 24% polyurethane from SNT Co. Ltd., Seoul, Korea. The structure was made by co-weaving spandex with polyester. It is very resilient, withstands harsh levels of wear and tear, is waterproof, and wrinkles less. SWCNT inks (0.1 wt%) were obtained from Jeio Co. Ltd., Incheon, Korea, and stretchable silver pastes were obtained from Dycotec Materials Ltd., United Kingdom.

As shown in Figure 1a–c, the fabric was immersed in an SWCNT ink, and squeezed by an automatic dipping/squeezing machine at the roller speed of 1.0 m/min and the cylinder pressure of 0.3 Mpa. This process allows SWCNT particles to penetrate well, and adhere to the surface of fibers. The excess water was removed by a two-way drying machine (Figure 1d) at 180–200 °C, for 10 min, and a motor speed of 1500 rpm. At the end of this step, we obtained SWCNT sensors (Figure 1e).

After that, we printed silver pastes onto the SWCNT fabrics by screen printing technologies at 30 mm/s and drying them for 12 min to remove the water again (Figure 1f,g). These steps are the structure–approach to create fabric strain sensors with a high performance. As described in Figure 1h, we obtained Ag/SWCNT sensors with a higher performance than the SWCNT sensors.

Finally, the sensors were shaped by a laser cutting machine to create arbitrary and customizable individual shapes (Figure 1i). In Figure 1j, the unique shape of the sensors was defined through many experiments. This step is the shape approach, which sharply increases the performance of the sensors.

## 3. Results and Discussion

### 3.1. Structure of the Sensors

**Figure 2:**
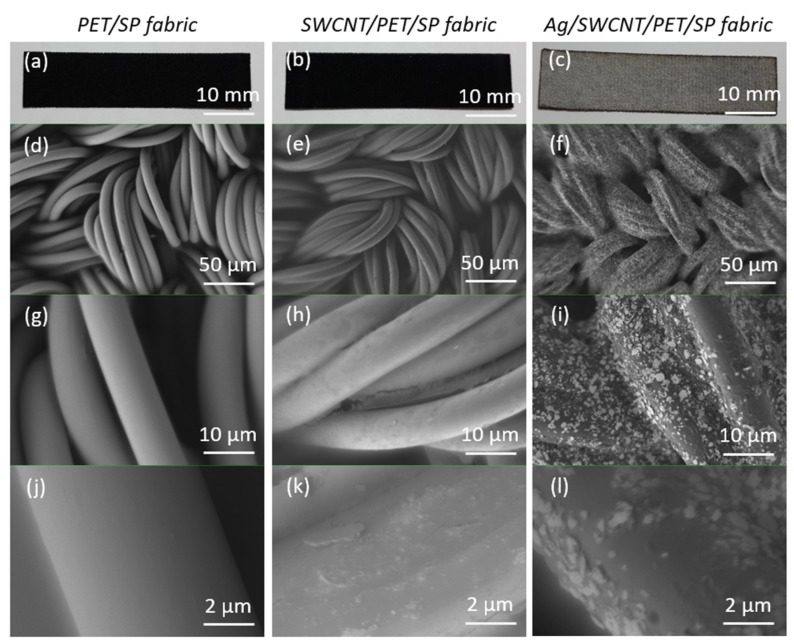
SEM pictures of the sensors at different magnifications consisting of (**a**) initial PET/SP fabrics at 10 mm, (**b**) SWCNT sensors at 10 mm, (**c**) Ag/SWCNT sensors at 10 mm, (**d**) PET/SP fabrics at 50 µm, (**e**) SWCNT sensors at 50 µm, (**f**) Ag/SWCNT sensors at 50 µm, (**g**) PET/SP fabrics at 10 µm, (**h**) SWCNT sensors at 10 µm, (**i**) Ag/SWCNT sensors at 10 µm, (**j**) PET/SP fabrics at 2 µm, (**k**) SWCNT sensors at 2 µm, (**l**) Ag/SWCNT sensors at 2 µm.

Figure 2 shows the structure of the sensors at different steps of the proposed process by scanning electron microscopy (Gemini SEM 300, ZEISS Corp., Germany). Magnification views are 10 mm, and 50, 10, and 2 µm, respectively. The filaments have a diameter of 10 µm, and appear loosely twisted with ample free space between the microfiber bundles. SWCNT particles are stuck randomly onto PET/SP fibers and can be observed as thin coatings with about an 80% coated rate, and Ag pastes covering CNT yarns on about 95% of the printing areas. The working principle of these sensors is based on the relationship between the mechanical (strain force) and electrical properties (Ag/SWCNTs). When loading a strain pressure, cracks will begin and propagate in the thin conductive layer (Ag/SWCNTs). Following this, the resistance will increase during continuous mechanical straining. In another instance, the edges of the cracks will reconnect when releasing the strain pressure. At this point, the initial electrical resistance of the sensors will be recovered. Based on the dimension, the resistivity of the composite (about 0.04 Ω/cm) is calculated by Equation (1), where ρ is the resistivity, R is the electrical resistance, A is the cross-section, and l is the length of the sample.

(1) $$\rho =\;\frac{R*A}{l}$$

**Figure 3:**
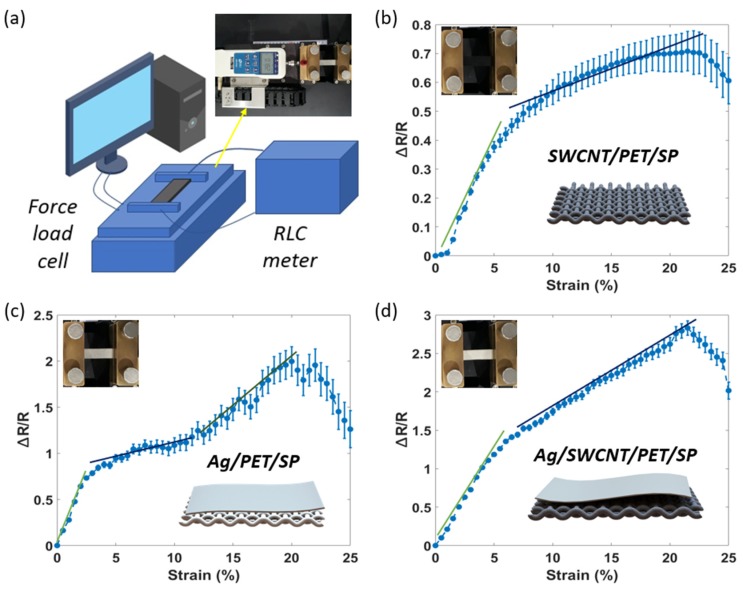
(**a**) Universal testing machine (UTM), (**b**) relationship of strain–resistance of SWCNT sensors, (**c**) relationship of strain–resistance of Ag sensors, and (**d**) relationship of strain–resistance of Ag/SWCNT sensors.

To characterize the electrical response of the sensors applied to the strain force, we developed an experimental setup to collect sensor data, using a customized universal testing machine (UTM) (Figure 3a) from Dacell Co. Ltd., Seoul, Korea, and a source meter (Keysight B2902A), as well as a gauge factor (GF) used to evaluate the sensitivity of the sensors. The GF can be determined through Equation (2), where R is the initial resistance, ∆R is the absolute change in resistance, L is the initial length, and ∆L is the absolute change in length.

(2) $$GF=\;\frac{∆R/R}{\varepsilon }=\;\frac{∆R/R}{∆L/L}$$

Five samples of each type (SWCNT, Ag, and Ag/SWCNT) at the same size of 5 x 1.25 cm in the total area of the sensors are used and averaged for characterization. The lowest sensitivity is the SWCNT sensor (Figure 3b), where GF = 4.5 at 20% of the strain. The Ag sensors have a higher sensitivity than the SWCNT sensors, where GF = 10 at 20% of the strain. However, the resistance change is unstable, as shown in Figure 3c. The highest sensitivity is the Ag/SWCNT sensor (Figure 3d) where GF = 20 at the strain under 5%, and GF = 12.5 at the strain from 5–20%. The error bars also demonstrated that the variability of data is highest at the SWCNT sensors and lowest at the Ag/SWCNT sensors. It is clear that the composite structure guarantees a constant and robust conductivity of the sensors under tension. We observed that the sensitivity of sensors would be reduced when the tension is over 20%. This can be explained by the variation of the distance between Ag/SWCNT particles based on the fabric’s structure. The initial increase in resistance was due to the increased length; the gaps between the Ag/SWCNT particles on the PET/SP fiber were also increased. However, in the decreasing resistance phase (over 20% of the tension), the gaps between the parallel fibers (PET/SPs) were reduced, allowing better contact and adding parallel conductivity paths, which resulted in a lowered resistance. This result likely depends on the structure of the fabric used, which limited the range of the stretchability. The stretchability of this fabric’s structure could be broken when the tension is over 20%. Of course, this point will limit some applications at special places that need a large amount of stretchability (such as the elbow, knee, etc.). In order to extend the working range, we suggest using highly stretchable fabrics. But this method will also change the characteristics of the sensors and requires more experimentation.

### 3.2. Shape of the Sensors

We determined the shape of the sensors as the best approach to improve sensor performances without increasing the manufacturing costs. Therefore, a series of tests were performed to collect the perfect shape of the sensors in common tension applications. There have been three rounds of experiments, consisting of (1) general shapes, (2) unique shapes with different numbers of buffer cutting lines (BCLs), and (3) unique shapes with different lengths of buffer cutting lines (BCLs).

**Figure 4:**
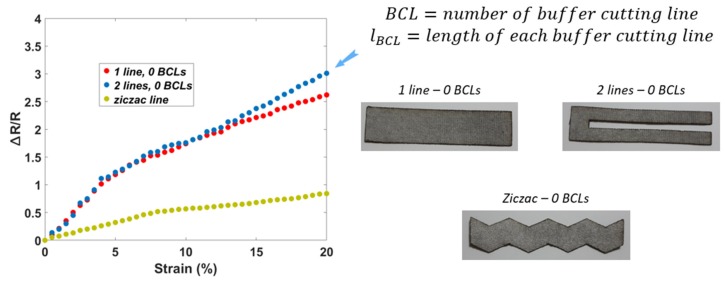
The first round in optimizing sensor performances based on different shapes.

Figure 4 shows the results of the samples in the first round. The best sensitivity (GF = 15) is of the samples with two sensing lines. Samples with triangular noses have a low sensitivity at GF = 4. Meanwhile, samples with one sensing line have GF = 12.5. The samples with two sensing lines show better sensitivity than the single sensing lines. Based on the space used to integrate sensors, we can choose the single-line or double-line sensor. For example, the wearable applications, which have a large area (on the arm, chest, thigh, knee, etc.), should integrate the sensors with multi-sensing lines to increase the performance or stretchability. In the case of a small space (on the finger, pharynx, etc.), the strain sensor with two sensing lines is difficult to use. In the context of this research, we monitor the movement of small parts of the body. Thus, the shape with one sensing line is selected for the next experiments.

**Figure 5:**
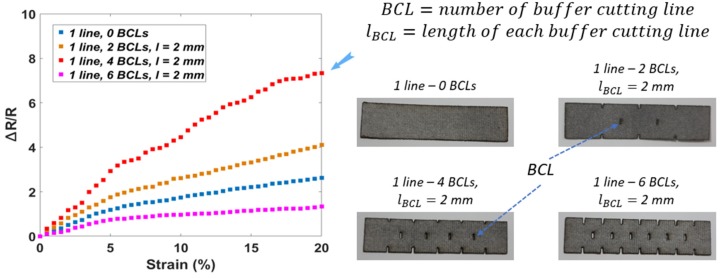
The second in optimizing sensor performances based on the different number of BCLs.

In the second round, samples with different numbers of BCLs (0/2/4/6 BCLs in the centerline of the sensing area) were investigated. As shown in Figure 5, it is clear that the BCLs inside the sensing area will improve the performance of the sensors. This could be explained as follows: the BCLs would increase the stretchability of the sensor leads so that the cracks appear easily on the conductive layer when tension is applied. Due to this, the change in resistance becomes faster and larger than the initial sensor (no BCLs) at the same percentage of tension. However, a small number of BCLs will not greatly increase performance, while a large number of BCLs will reduce the connectivity of Ag/SWCNT particles (Table 1). Here, the sample with four BCLs inside the sensing area has good performance (GF = 36.25). The GF of other samples is 20.45 with two BCLs and 6.67 with six BCLs, respectively.

**Figure 6:**
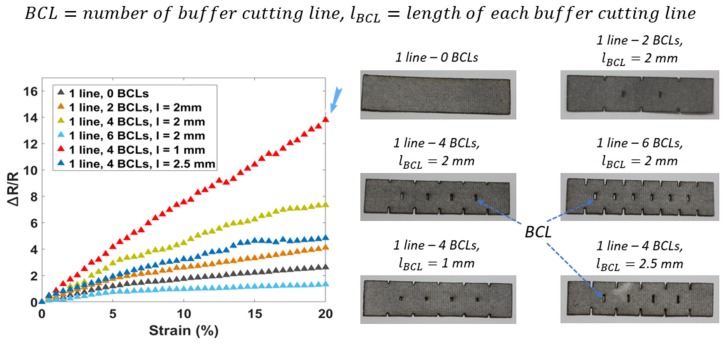
The third round in optimizing sensor performances based on the different lengths of the BCLs.

Figure 6 shows the final experiments with different lengths of the BCLs, consisting of L_BCLs_ = 1, 2, and 2.5 mm. The results of the samples with small BCLs (1 mm) show a fantastic sensitivity (GF = 71.5), more significant than six times when compared to samples without any BCLs. In other words, the large length of the BCLs (2.5 mm) will decrease the performance of the sensors (GF = 23.5).

**Figure 7:**
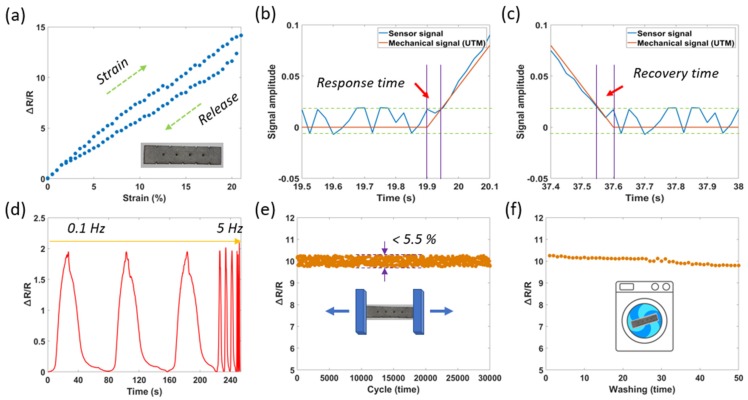
Characteristics of the Ag/SWCNT sensors (integrated small BCLs) consisting of (**a**) hysteresis, (**b**) response time, (**c**) recovery time, (**d**) signals at different frequencies, (**e**) durability after 30,000 stretching/releasing cycles, and (**f**) durability after 50 washing times.

We investigated the hysteresis of sensors to evaluate usability in practical applications (Figure 7a). Once again, the Ag/SWCNT sensors (integrated small BCLs) show a better hysteresis when the maximum hysteresis is 10% for the applied strain at 20%. Meanwhile, the maximum hysteresis of SWCNT sensors is 20%. Hysteresis of the Ag/SWCNT sensors (without BCLs) is slightly lower than the Ag/SWCNT sensors (integrated small BCLs), but with little change.

The response time was calculated as the time span between the mechanical stimulation of the universal testing machine (UTM) and the sensor signal when loading at a speed of 1 mm/s. As described in Figure 7b, a fast response time of less than 50 ms was observed in the increasing step of the tension. We also evaluated the recovery time (relaxation time) of the sensor in the same conditions. Figure 7c showed a short recovery time of less than 60 ms in the decreasing step of the tension. Fast response and recovery times are important values to prove that the sensor can catch up with the change of the applied strain.

Attributed to the high recovery performance of the Ag/SWCNT cracks, the sensors have a stable response under a wide mechanical frequency range from 0.1 to 5 Hz, as described in detail in Figure 7d. In order to evaluate the dynamic durability of the sensors, we determined the stable electrical functionality and mechanical integrity during loading/releasing cycles. The experiment performed by the UTM machine and the resistance was measured at every 50 cycles (at the strain of 15%). We observed that the uniform resistance changes of less than 5.5% were recorded after 30,000 cycles, as described in Figure 7e. In the washing test, the samples were checked using a mini washing machine (LG-W0082) from Daewoong Co., Seoul, Korea. Each washing time had a duration of 10 min, a squeezing time of 2 min, and a drying time of 7 min at 100 °C. The sensors demonstrated a small resistance change (at the strain of 15%) of less than 7% after 50 washing times (Figure 7f), indicating that the Ag/SWCNT sensors were highly reproducible. 

**Figure 8:**
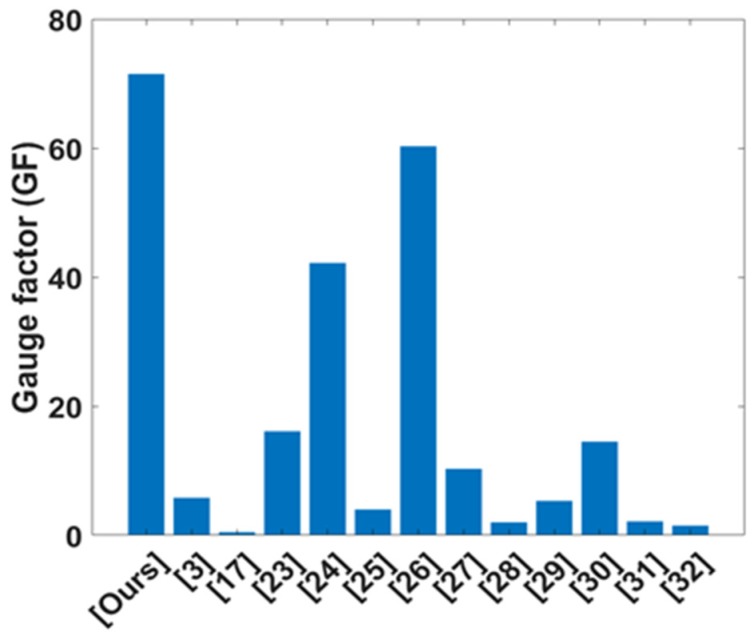
Comparison (GF) with other studies.

In an overview comparison, our sensors (with GF = 71.5) have higher sensitivity than other studies [3,17,23,24,25,26,27,28,29,30,31,32] at 20% of the tension. As shown in Figure 8, the GFs of reference researches are lowest at 0.46 [17] and highest at 60.3 [26], respectively. The manufacturing processes of some studies are complex [26,28,31], and they are not easily translated to mass-production or industrial applications.

## 4. Applications of Sensor

**Figure 9:**
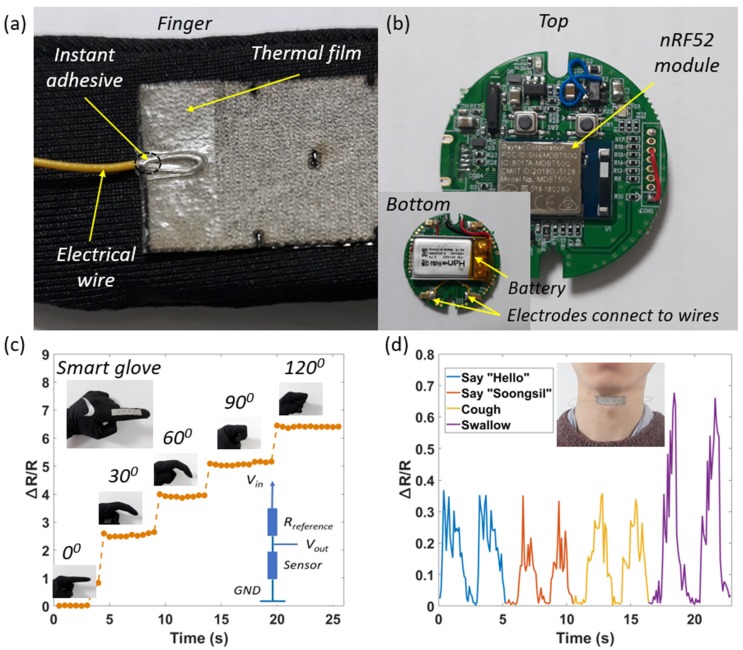
(**a**) structure of sensors on fingers, (**b**) hardware platform, (**c**) signals of finger motions at different angles, and (**d**) signals of pharynx motions when speaking, coughing, and swallowing.

To demonstrate the potential for the Ag/SWCNT strain sensors (integrated BCLs) for use in a practical application, the proposed sensor was attached to a glove for finger motion at different angles. As described in Figure 9a, an instant adhesive was used to fix the position of the electrical wire (AWG 32). A thermal film was used to ensure the connection between the electrical wires and the electrodes of the sensors [9]. This is a 100 µm elastic thin film with 100% polyurethane (PU) from Sealon Ltd., Seoul, Korea. The hardware platform is an integrated circuit (Figure 9b), consisting of an nRF52 module (a combination of microcontroller, Bluetooth, etc.) [33], and a lipo battery (3.7 V). Using a voltage divider circuit, the electrical resistance variation of the sensor is converted into a voltage variation. It is calculated to take about 0.01 s (10 ms) to read an input signal, and a maximum reading rate is about 100 times per second. Once the finger is bent, a tension force is applied to the sensor and increases the resistance. As shown in Figure 9c, the resistance change will be at its maximum at 120 degrees. Signals will be recorded and transmitted to a mobile phone/laptop via a Bluetooth connection. In the other exciting application, by attaching the sensor to the neck, pharynx motions when speaking, coughing, and swallowing exhibited obvious and consistent signals (Figure 9d). However, the sensor had a small limitation in respiratory monitoring, and the recorded signal was unclear. In this case, we suggest using an artificial intelligence (AI) model to classify the responding signals to achieve optimum accuracy. This also is the direction of the research in the future of the project.

## 5. Conclusion

In this work, we have proposed a two-step approach in the fabrication and optimization performance of e-textile strain sensors. The combination of silver paste and SWCNTs has improved the sensitivity of typical SWCNT sensors. Notably, the unique sharp shape of small BCLs through laser cutting has increased the GF of sensors up to six times (from 12 to 71). A quick response (< 50 ms) and excellent stability (> 30,000 cycles) also demonstrated the usability of sensors in practical applications. More importantly, the fabrication process showed a batch manufacturing ability that could be easily applied in the industry. Finally, we demonstrated two applications of sensors for detecting the angle of finger motions and the movements of the pharynx when speaking, coughing, and swallowing. The above advantages provide potential in the field of smart e-textile sensors in human motion monitoring, healthcare, or soft robotics.

## Figures and Tables

**Table 1:** The GF of Ag/SWCNT samples at the strain of 20%.

The GF of Ag/SWCNT samples at the strain of 20%	
Sample	GF
Zizac line - 0 BCLs	4
2 lines - 0 BCLs	15
1 line - 0 BCLs	12.5
2 lines - 2 BCLs, $l_{BCL}$ = 2 mm	20.45
1 line - 4 BCLs, $l_{BCL}$ = 2 mm	36.25
1 line - 6 BCLs, $l_{BCL}$ = 2 mm	6.67
1 line - 4 BCLs, $l_{BCL}$ = 0.5 mm	25.6
1 line - 4 BCLs, $l_{BCL}$ = 1 mm	71.5
1 line - 4 BCLs, $l_{BCL}$ = 2.5 mm	23.5
